# Editorial: Psychological Factors as Determinants of Medical Conditions

**DOI:** 10.3389/fpsyg.2019.02502

**Published:** 2019-11-08

**Authors:** Gabriella Martino, Viviana Langher, Valentina Cazzato, Carmelo Mario Vicario

**Affiliations:** ^1^Department of Clinical and Experimental Medicine, University of Messina, Messina, Italy; ^2^Department of Dynamic and Clinical Psychology, University of Rome “Sapienza”, Rome, Italy; ^3^School of Natural Sciences and Psychology, John Moores University, Liverpool, United Kingdom; ^4^Department of Cognitive Sciences, Psychology, Education and Cultural Studies, University of Messina, Messina, Italy

**Keywords:** psychological factors, adherence, compliance, anxiety, stress, quality of life, emotional distress, chronic diseases

Life expectancy is increasing world-wide and age-related diseases are becoming a major health concern. It is known that chronic diseases and related outcomes may seriously impact people's perceived quality of life, and this effect may in turn lead to psychopathological consequences. Indeed, psychopathological symptoms frequently occur in chronic medical conditions and can even predict and impact mortality independently of a wide range of potential confounders.

This Research Topic includes interdisciplinary and multidisciplinary contributions in order to understand how psychopathological aspects may seriously impact somatic symptoms and medical outcomes, especially in age related common chronic diseases. The scientific interest in understanding how psychological factors may determine several medical conditions is dramatically growing. In keeping with this awareness, the current Research Topic aimed to provide a significant contribution to this field by inspiring the submission of scientific articles promoting a multi/inter-disciplinary approach, and suggesting a new direction in psychopathological research and prevention, leading to screening subjects at risk for medical events in order to individualize and improve diagnostic and therapeutic approaches. Our collection includes 15 research articles that explore the reciprocal link between psychological determinants and medical conditions with regard to three fundamental domains: cognition, stress, and emotion. Five articles, particularly, explored the influence of ADHD (Klein et al.), metabolic syndromes (Guicciardi et al.; Marchini et al., 2018; Settineri et al., 2019), early life stress (Péterfalvi et al.), viral infections and mental health issues on cognitive processes; five articles explored the influence of stress on metabolic syndromes (Kelly et al.; Martino et al.), quality of life in liver transplant recipients (Funuyet-Salas et al.), autoimmune diseases (Cataudella et al.), mental health of workers, perceived pain during upper endoscopy (Lauriola et al..), and fibromyalgia and rheumatic diseases (Marchi et al.); lastly, three articles provided a contribution to the link between emotion processing and/or mood diseases in psoriasis (Ciuluvica et al.), psychosomatic disorder (Settineri et al.), and pain therapy (Fiegl et al.).

Much more effort needs to be done and many issues remain to be addressed to boost our knowledge with special regard to psychological factors as determinants in the setting of medical disease (Mangelli et al., 2005). Overturning the usual causal direction body-mind, evidence exists regarding the key role of psychopathological factors in the history of chronic illness. It is known in fact that a strict evaluation of the psychological variables could contribute to a better understanding of the individual condition and possibly predict the risk of onset of new medical diseases or complications. Several studies have emphasized, in chronic diseases, the neuropsychological functioning in chronic diseases (Catalano et al., 2019), even in ADHD (Martino et al., 2017; Fabio et al., 2018; Salehinejad et al., 2019) and it is known that the neuropsychological evaluation may reflect also the involvement of the frontal lobe functions (Bechara and Noel, 2010; Vicario and Martino, 2010). It could be furthermore interesting to provide more work on the mechanisms underlying the relationship between clinical psychological symptoms as anxiety, depression and health related quality of life, and chronic medical conditions among which we can consider metabolic, bone, celiac, and thyroid diseases (Misra and Lager, 2008; Di Corrado et al., 2013; Smith et al., 2013; Castelnuovo et al., 2015; Del Piccolo et al., 2015; Catalano et al., 2017, 2018; Guicciardi, 2017; Le Donne et al., 2017; Martino et al., 2018a,b, 2019). It would be also interesting to investigate how negative emotions, such as anger and disgust, are linked to different psychopathological conditions, such as depression and personality disorders (Vicario, 2013; Craparo et al., 2016; Vicario et al., 2017). It would be also exciting to explore how the treatment of psychological factors determinants of several medical conditions can be enriched by different therapeutic approaches, including the use of non-invasive brain stimulation technologies, which are known to be effective for the treatment of psychopathological conditions (Vicario and Nitsche, 2013a,b; Gangemi et al., 2018; Vicario et al., 2019).

These examples from the studies of this Research Topic are representatives of many endeavors that may deepen our understanding of the link between psychological factors and medical conditions. In conclusion, we seek to advance the knowledge with special regard to the psychological factors as determinants of medical conditions by highlighting specific psychological characteristics which may facilitate prevention, intervention approach and plans. Moreover, we trust that deepening our understanding of these topic may help researcher and clinicians to develop prevention strategies which will improve mental health and quality of life.

It has been a great pleasure and honor to be involved in this Research Topic. We would like to thank all the Authors, Reviewers, and the entire Frontiers Editorial and Developmental Staff for helping and assisting to make this Research Topic possible. Our satisfaction leads us to look forward with pleasure and interest to further address the links between psychological factors and medical conditions in future work.

## Author Contributions

GM and CV wrote the first draft of the manuscript and revised it critically. VL and VC provided opinions on it. GM, CV, VL, and VC read and approved the submitted version.

### Conflict of Interest

The authors declare that the research was conducted in the absence of any commercial or financial relationships that could be construed as a potential conflict of interest.
